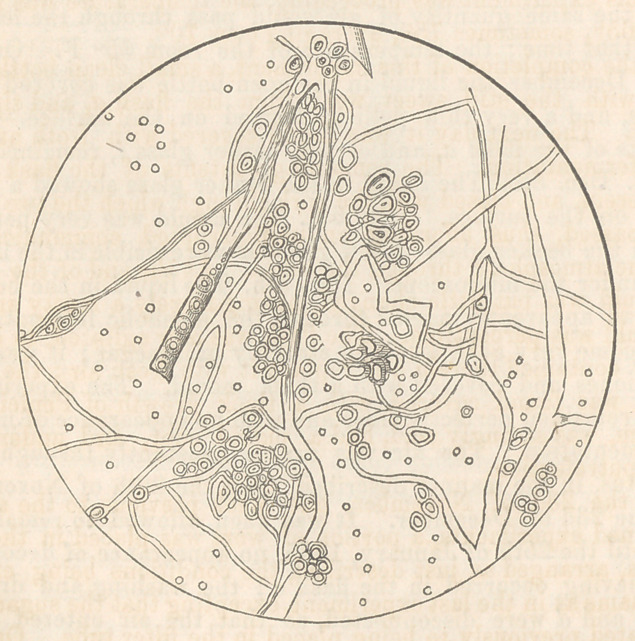# An Investigation into the Facts and Theories of Fermentation and Putrefaction

**Published:** 1855-05

**Authors:** Henry Pemberton

**Affiliations:** Practical and Analytical Chemist; Philadelphia


### MEDICAL EXAMINER.

NEW SERIES.—NO. CXXV.—MAY, 1855.

### ORIGINAL COMMUNICATIONS.

*An Investigation into the Facts and Theories of Fermentation and Putrefaction.* By HENRY PEMBERTON, Practical and Analytical Chemist.

Three classes of phenomena, closely allied, if not identical with each other, attract our attention at every step. These are, spontaneous decomposition or eremacausis, fermentation and putrefaction. It is most probable that no essential distinction exists between these phenomena, our imperfect knowledge preventing us from tracing the action that takes place to its source; but enough is known to justify the belief that they are but the varied modes of action of the same principle, governed and influenced by the same laws, and having their origin in the same ultimate cause. The diversity of composition, the variety of conditions, and greater or less intensity of action, are sufficient to account for the difference in the processes and products of organic decomposition.

The term eremacausis, or slow combustion, is principally confined to that class of decompositions in which the decomposing material seems to moulder away, apparently unaccompanied by infusorial or cryptogamic vitality, without emitting offensive gases, and without producing visible secondary products, and leaving as the final residue merely the so-called mould, (humus or humic acid) mixed with the saline constituents of the substance.

By fermentation is generally understood those peculiar decompositions of organic matters, at common temperatures, originating either spontaneously, or under the influence of another portion of a substance of similar character then undergoing a like change, the ultimate products being for the most part solid or liquid bodies of a less complex organization, and possessing a structure still further removed from the immediate formations of animal or vegetable life. These products are usually unaccompanied by offensive gases, and still possess a highly organized structure, so as to render it impossible to obtain them from the direct combination of their elements, or from other than higher organized bodies than themselves.

By putrefaction, we understand those decompositions that are attended with the evolution of offensive gases, consisting of phosphuretted and sulphuretted hydrogen, hydrocarburets, carbonic acid, ammonia, and the fixed and volatile salts thereof, together with certain volatile organic bodies to which the disagreeable odor is in great part due, and of whose history little is as yet known. The subjects of this change are principally the protein bodies, or substances that, like them, are rich in nitrogen, sulphur, etc., the presence of which give rise to the character by which this mode of decomposition is distinguished from others.

All these phenomena agree in requiring certain conditions that are essential to their existence. These are, warmth, moisture, and the presence of oxygen or atmospheric air ; to which is to be added, in the opinion of many writers, the presence of the sporules or ova of microscopic organisms.

The importance of the rôle played by these phenomena in the economy of nature, is secondary to none. To them we are indebted for the restoration to the treasury of the elements, of those effete tissues and worthless frames that once were the dwelling places of animal or vegetable life, but which would now lie as useless incumbrances upon the soil, excepting so far as they might furnish food to other creatures—an outlet too limited and too uncertain, to meet the wants of Providence. By these means, however, no sooner does a flower wither, or an animal die, than the processes of decomposition commence, and in proportion to the urgency of the wants of the young and growing vegetation, stimulated by the warmth of the early summer sun, is the rapidity with which these agents work; dissolving the combinations now rendered useless, and throwing off their elements in the form of compounds, nauseous to man, but doubtless filled with sweet odors to the tender plant.

The position occupied by these agents among the dynamical powers of nature is as interesting as anomalous. Only finding a dwelling in those places where vitality has dwelt before, they seem a species of life in death, imitating, in a feeble manner, those processes that were carried on when life itself was there. To the philosopher they offer the only point where he can observe anything resembling the transitive steps from the inorganic to the organic world; where he can watch, as it were, in their rudimentary form, those curious combinations and changes that take place within the living body, and find, if man is ever permitted so to do, the key to the mystery of vitality.

Whatever may be the variety of opinions held with regard to the immediate exciting causes of malarious diseases, there can be no doubt as to the close and intimate connection of malaria with the presence of decomposing vegetable matter. Dr. La Roche, in his most admirable work upon pneumonia and malaria, proves most satisfactorily that organic matter in a state of decomposition is present in all cases where miasma originates; and although the conditions of heat and moisture are essential, the presence of decaying vegetation is also indispensable.—Since it is most probably only by the investigations of the obscure phenomena of fermentation that we can ever hope to arrive at a knowledge of these occult influences, this process becomes of the greatest importance, and deserves a far greater share of attention than it has as yet received.

Before considering the history of the laws that govern fermentation, it is indispensable that we should be in possession of all the important facts relating to the subject, as it is only by the close comparison of fact with fact that this branch of science can ever be removed from the obscurity in which it still remains. No department of knowledge has received less attention, it being generally considered as beyond the reach of explanation, and, like the doctrine of catalysis, as being the "ultima thule" of investigation. Thus a German writer, of high position, (Professor Julius Schlosberger,) who has himself paid much attention to this subject, in speaking of the action of certain animal poisons,* uses the following language: "Since all the theories of the nature of the poison, capable of experimental proof, are not yet exhausted, I think it best *to discard the idea of a ferment, since its nature prevents all further investigation.*" Such are the views generally held regarding this process.

We will now endeavor to present, in as concise a form as possible, all the facts and experiments that we can find, that may cast any light upon the process or theories connected with this subject. In every case we have referred to the original paper by the author, when obtainable, and not to a mere abstract by another, as errors and misstatements frequently occur in copies and abridgments, and many points are rendered obscure which are perfectly intelligible when in connection with the rest of the text.

In a substance undergoing eremacausis, the carbon and hydrogen unite with oxygen, forming carbonic acid and water. The nitrogen is evolved partly as gas, partly in the form of nitrous and nitric acids. The fixed residue from wood, etc., humus or humic acid, is very rich in carbon, most of the oxygen contained in the wood uniting with the hydrogen and part of the carbon, the rest of the carbon being ultimately, though very slowly, oxidized by the atmosphere. The hydrogen of organic substances, when the supply of air is limited, unites with the nitrogen of the atmosphere, forming ammonia, thus affording the nitrogen necessary to the growth of the mould plants on the decomposing substance.† Frequently, when the access of air is impeded, while the other conditions remain the same, eremacausis ceases, the formation of mould commences, and true putrefaction sets in.† A substance undergoing eremacausis, induces change in other bodies, the union of hydrogen and oxygen, etc. (Saussure.‡) During eremacausis, heat is evolved, generally scarcely perceptible, but in favorable circumstances producing actual ignition. Frequently eremacausis is induced in a substance otherwise incapable of it, by contact with a body in that condition. Thus, decaying wood causes the oxidation of diluted alcohol into acetic acid, and other decaying matter will produce

*Schmidt's Jahrbuch, 78, 4. † Gmelin's Handbuch.
‡ Liebig's Handwörterbuch der Chemie.

the oxidation of hydrogen into water, and of ammonia into nitric acid. These substances unite with oxygen at common temperatures, only in the presence of decaying organic matter, the action ceasing as soon as the decomposing substance is removed.*

It has been observed by the colonists in America, that a large tree, when felled, completely disappears in from 33 to 35 years, with the exception of the bark; the leaves, small twigs, etc., of course in a much shorter period.* The dry rot of timber may perhaps be considered as an instance of eremacausis, although frequently attributed to the effects produced by a vegetable parasite that attacks it. The author has seen four floors of a large store, constructed in the strongest manner, completely destroyed within two years by this cause, so that the heavy joists would not bear their own weight. In this case the timber was covered with a green mould, quite perceptible to the naked eye. Improper ventilation and a certain amount of dampness appear to be essential conditions.

When caseine, free from sugar of milk, is covered with water and exposed to the air for two or three months, it is converted into ammonia, valerianic acid, butyric acid and leucin; a volatile crystallizable solid, having the odor of fæces, and an acid convertible into tyrosin and ammonia, are also formed. Gluten, which is identical with caseine, yields similar products. Bile, similarly treated, undergoes two stages of putrefaction; in the first are formed choloidic acid, taurine and ammonia; in the second, cholinic acid. These metamorphoses of caseine and of bile are identical with the products obtained by the action of powerful chemical agents upon them, and are in no way peculiar to the process of putrefaction. Moist fibrin, exposed in an open glass vessel and in contact with water, quickly putrefies. The change commences on the surface and is propagated downwards, the upper part becoming grey and soft, while the lower part remains white and firm. By frequent agitation the change is much accelerated. The fibrin of blood, covered with water and exposed to the air until it liquefies, yields a fluid having the properties of albumen, forming a coagulum by heat resembling that from white of egg.* Of various animal matters, the brain, muscles, liver, spleen and other glands, pu-

* Liebig's Handwörterbuch.

trefy first, and develop carbonic acid even in the first hour. The skin, hard portion of the brain, intestines, veins, arteries and stomach, do not develop carbonic acid under 24 hours. The sinews, intervertebral cartilage, etc., undergo no change for a much longer period. When putrefaction has once commenced, it proceeds with the rapid absorption of oxygen and the generation of carbonic acid and ammonia; sulphuretted hydrogen and carburetted hydrogen (marsh gas) are frequently found, particularly in the putrefaction of the muscles, (J. Davy.*)

When putrefying blood, caseine, etc., are mixed with a solution of sugar, the disagreeable odor is at once diminished, and as soon as fermentation has fully set in, ceases entirely. The rapidity and products of putrefaction depend upon the nature o the atmosphere surrounding the substance. When fresh beef is enclosed in an atmosphere of oxygen gas, it becomes a brighter red on the first day, then pale and moist; drops of clear liquid sweat out, which afterwards become milky, and in 11 days it is putrid. In 51 days it liquefies with an unbearable odor. A large portion of the oxygen is converted into carbonic acid. In hydrogen gas the meat becomes brown, is not putrid in 11 days, and has a slight sour smell. In 54 days, the meat preserves its appearance, but has an intolerable odor, yet differing from that in oxygen; carbonic acid was found mixed with the hydrogen. In carbonic acid it becomes first brown, then red, then pale, looks like cooked meat; is soft, but not glutinous; dries in the air, without spoiling after 51 days. In sulphurous acid gas the meat is at once discolored, is much harder and drier, and after 76 days dries in the air within four days without decomposition. The same effect is produced by fluosilisic acid gas. In oxide of nitrogen it becomes immediately bright red; after 11 days it is still red, inodorous, and dries in the air. After being 134 days in the gas it is still of a fine red color, firm, and smells of nitric acid. In ammonia, it remains 76 days without change; when taken out it is soft, without odor, and dries in the air without spoiling, into a shiny brown mass, (Hildebrandt.*)

Pieces of meat placed in contact with plates of copper and of zinc do not enter as soon into putrefaction; those pieces in contact with the zinc become electro-negative, and give ammoniacal

* Gmelin's Handbuch.

products and hydro-carburets (marsh gas?), those in contact with the copper plates give rise to the formation of acids, acetate of copper, etc., (Charles Matteuci.*) According to Wurtz,† fibrin when exposed to the air for some time is converted almost entirely into butyric acid. Albumen as well as fibrin is convertible, under certain conditions not very well known as yet, into a peculiar fatty body called adipocire, consisting of the fatty acids saponified by ammonia, mixed with variable proportions of the free acids. It is frequently formed in bodies that have been buried in a damp soil, or immersed in water containing much carbonic acid, occasionally constituting the sole residue, the bones even having disappeared. According to C. Blondeau‡ it can be formed from fibrin under the same conditions that attend the conversion of caseine into the so-called Roquefort cheese, and is attended with the growth of a green cryptogamic plant, to which he applies the name of Torula viridis, the presence of which he considers essential to the process.

When fresh milk is placed in vessels of the same form, but constructed of different materials, the coagulation takes place at different periods. Thus on the 21st of April milk was boiled and placed in vessels of various kinds with the following results:

On the 24th, coagulated in the vessels of porcelain, glass, and lead.
" 25th, " " Platinum, solid and tinned iron.
" 26th, " " Tin, bismuth and antimony.
" 27th, " Sulphur. On the 28th, in zinc.
" 30th, " Copper and brass, after being covered with mould.

The odor and taste of the curd and the character of the cryptogamic growth, also differed with the material of the vessels, (M. Bouchardat.§) When milk is boiled in a bottle which it three fourths fills, and the air excluded, it is found at the end of 20 days in June, to preserve its properties unchanged. The enclosed air still contains 16.7 per cent. of oxygen. If the bottle is only $\frac{2}{3}$ full or less, the oxygen is absorbed and carbonic acid formed; the milk remains fluid. When only $\frac{1}{4}$ full, the milk has an acid reaction, coagulates

* Annales de Chemie et de Physique, t. 42, 1829.
† Annales de Chem. et de Physique, t. 11, 3e. s. 1844.
‡ Jour. de Pharmacie, t. 12, 3e. S. 1847.
§ Jour. de Pharm., t. 19, 1833.

on heating, and yields a little alcohol on distillation. (Th. v. Dusch and Gmelin.*) Schröder and Von Dusch,† in their experiments upon the filtration of air, found that milk soured in a flask after boiling, although filtered air only had access thereto, while meat broth remained, under the same circumstances, unchanged.

Liebig states that milk placed in a vessel and covered with paper, undergoes the lactic fermentation without the production of vegetable growth. When milk is exposed under ordinary circumstances to the air, it rapidly coagulates and becomes sour. Coagulation is also effected by immersing in the milk a piece of rennet, (the mucous membrane of a calf's stomach,) or by the addition of water in which the rennet has been macerated. This effect, however, is only produced after the membrane has been in contact with water and air for some hours, perfectly fresh rennet being without action upon milk. The curd and whey thus obtained differs from that formed by the spontaneous coagulation of milk, in possessing an alkaline or neutral reaction, while that formed without rennet is strongly acid. The curd is also insoluble in carbonated alkalies, excepting by long boiling, and contains therein in chemical combination the phosphates of lime and of magnesia, the presence of which render it insoluble in alkaline solutions. As an article of diet, therefore, whey from sour milk is preferable to that produced by rennet, the phosphates so necessary to the growth of the body being absent in the latter. Rennet, capable of coagulating milk, causes the conversion of sugar into lactic acid, but if left exposed to the air for a long time excites the alcoholic fermentation instead.‡ A mixture of caseine, sugar and water exposed to the air for some time becomes covered with various species of cryptogamic vegetation. The most prominent is the Penicillium glaucum, then a plant formed by the union of globules into twigs (Mycoderma vini), another covered with green sporules, and an orange red cryptogamia, resembling the Oidium aurantiacum. When whey from the spontaneous coagulation of milk is set aside at a temperature of 72° to 77° F. it becomes in a few days cloudy, and shows, under the

* Gmelin's Handbuch.

† Medical Examiner, vol. 10, and Liebig's Annalen, 1854.

‡ Liebig's Handwörterbuch.

microscope, a great number of globules. In 24 hours, a pellicle covers the surface, in which can be seen the stems and branches of the Penicillium glaucum, soon giving rise by their growth to a vegetation of regular form, consisting of twigs, radiating from a centre. After a month, on adding alcohol to the liquid, the globules coagulate, and form a mass resembling gum, white at first, but soon becoming yellow; much lactic acid is present. (C. Blondeau.*)

The manufacture of Roquefort cheese has attracted much attention, from the curious fact that it can only be made in the village of Roquefort, whence it derives its name. This peculiarity is owing to the nature of the caverns in which the cheese is placed to ripen. According to Marcal de Serres,† who paid a visit to the place, for the purpose of examining into its manufacture, these caverns constitute what are called ice grottos, and possess throughout the summer a temperature but little above the freezing point (from 41° to 45.5° F.) During warm weather a constant current of cold air issues from their mouths, but in winter the current is reversed. This effect is produced by the warm dry air, that enters through numerous fissures at the top of the caverns, being chilled by the damp rocks, becomes specifically heavier, and consequently descends. In its course downwards, it meets, constantly, in the narrow passages, with cold water, exposed in the manner most favorable to evaporation, which cools the air more and more, until it finally reaches a point at which it is fully saturated with vapor, when further reduction ceases. In cold weather, when the external temperature is lower than the rates here indicated, the action is reversed. The air in the caverns, being lighter than that outside, ascends, and is replaced by fresh air rushing into the lower apertures, thus producing the upward current. Caves possessing similar characters are found in various parts of this country, and are perhaps as well adapted to the manufacture of this cheese as those of Roquefort. Blondeau‡ states that these cheeses are made from the milk of sheep, coagulated by the mucous membrane from a lamb's stomach. The curd is kneaded and pressed

* Jour. de Pharm. 1847.

† Annals de Chemie et de Physique, t. 63. 1836.

‡ Jour. de Pharm. t., 12. 1847.

into form, being previously well mixed with pieces of mouldy bread. It is then placed in the caves above mentioned, which are not damp, but perfectly dark, where it remains about two months. When taken out, it is found to be covered with mould like that upon the bread. The caseine is converted into a fatty substance, like butter, but differing in its chemical relations. This change takes place from without, inwards, and is produced by the cryptogamic vegetation, which develops itself with great rapidity, the diffusion of the mouldy bread throughout the mass introducing the germs of the plant into every part. No fatty matter is contained in the cheese previous to its introduction into the caves, but when examined after fifteen days it is found in large quantity.

Blondeau also states that during the progress of the lactic fermentation, when chalk has been added to the mixture of caseine, sugar and water, to prevent the increase of free acid in the liquid, that a portion of the caseine unites with the chalk at the bottom of the vessel, and, when separated by muriatic acid, forms a fatty substance, that makes greasy stains on paper. At the end of a month, when the fermentation is ended, the compound of caseine with chalk is no longer found, the caseine being replaced by butyric acid.

Albumen that has been boiled for a long time is almost incapable of putrefaction. Membrane or fibrin that has been washed with alcohol or ether, does not enter nearly as readily into putrefaction, as it does if simply washed and left in contact with water,) probably from the alcohol destroying the organic germs present.) Nearly all other organic substances containing nitrogen, after exposure to the air for a length of time, are capable of inducing the lactic fermentation. Boutron and Fremy state, that fresh animal membranes exert no appreciable effect upon neutral substances, such as sugar, starch, etc., but after having been preserved in water in contact with air for some time, possess this power in the highest degree. According to Thenard, albumen can remain in contact with sugar and water for two months before it will induce its conversion into alcohol and carbonic acid (probably with exclusion of air.)

During the lactic fermentation, if the acid accumulates beyond a certain amount, it stops the fermentation ; if the acid is removed by saturation with an alkali, avoiding an excess, the fermentation recommences, and continues until the quantity of acid formed again interrupts it, or until the sugar is exhausted. Caseine is not capable of acting indefinitely as a ferment, but after the formation of lactic acid has ceased, it becomes capable of inducing the alcoholic fermentation.* Equal quantities of cane sugar requires eight times as much ferment to induce fermentation as grape sugar does; the latter is the only kind of sugar capable of entering into fermentation; the other sugars are converted into this before undergoing further change (H. Rose.†)

Bouchardat‡ states that the brain of an adult man acts as a powerful alcoholic ferment; while that of a newly-born animal, under the same circumstances, produces the mucous fermentation—probably owing to the unequal lengths of time that the brains had been exposed to the air, and not to any difference in composition.

The commencement of fermentation, and the first appearance of organic growth in a fermentable liquid, are accurately described by Quevenne.§ The phenomena are precisely such as the author has witnessed in the spontaneous change of fresh wort, (infusion of malted grain) at a temperature of 70° F.

"When a limpid fluid, containing the necessary elements for the development of fermentation, is exposed to the air at a temperature of 68° to 77° F., it soon becomes cloudy. If now examined by the microscope, an infinite number of small oblong bodies, and of little black points, isolated, or united in a lineal series, are seen. These objects all have a diameter at most of $\frac{1}{800}$ millemetre. At the end of a variable period, sometimes a few hours, sometimes several days, there appear globules, very pale at first, the terminal circle being very undecided, the centre colorless, united, isolated, or grouped in chaplets or little masses. These are the globules of the ferment. At the instant of their first appearance, they have already their usual size, and do not seem to increase in size during the phases of the fermentation."

* Sur la fermentation lactique, Jour. de Pharm. t. 27, 1841.

† Jour de Pharm., t. 27, 1841.

‡ Comp. rendus de l'Acad. de Sciences, 1838.

§ Jour. de Pharm., t. 4, 3e. s., 1843.

The description given of this process by Mitscherlich* agrees with the above, except that he has observed a small globule occasionally joined to a larger one, indicating growth by gemmation. He considers the globules different from those formed when a ferment is added.

When any fluid containing albumen in solution, is very slightly acidified, and set aside, it is found in a very short time to contain a number of very small globules, which were mistaken by Liebig for albumen precipitated in a globular form, but which subsequently was proved by Andral and Gavarret to be the first stage of the development of the globules of the Penicillium glaucum. These globules appear in fresh serum from blood, in serous discharges in various diseases, in the serum accumulated in the peritoneal sac, in the serum of hydrocele, serum from the effusion from a blister, and in serum obtained by filtration of pus, (the pus globule remaining on the filter, while the serum passes through.) These represent all the varieties presented by morbid secretions containing albumen. The globules form first in those parts of the liquid in contact with air. If the serum is placed in a flask, gently warmed, and the air expelled by carbonic acid or by hydrogen, and the flask then corked, no globules or other forms of vegetation appear, but on exposure again to the air, they rapidly develop themselves.

Turpin has found the same plant—the Penicillium glaucum—in the milk from diseased cows. The pellicle formed by the growth of this plant in albuminous fluids, will not produce the alcoholic fermentation when placed in a solution of sugar and water, nor even when the latter is mixed with serum, lactic acid always appearing.† Sugar, under the influence of certain animal matters, is sometimes wholly converted into lactic acid; at other times, with the same animal matters, and conducted in the same manner, very little lactic acid, but large quantities of mannite, a viscous substance, and occasionally even alcohol and carbonic acid, are formed. These effects are dependent upon the state of decomposition of the animal substance, varying with the length of time it has been exposed to the air. In the same manner

* Jour de Pharm., t. 24, 1838.

† Researches on the development of P. glaucum in normal and diseased fluids, by Andral and Gavarret; Ann. de Chem. et de Physique t. 7, 3e. S. 1843.

diastase, which transforms starch into dextrine and into sugar, when exposed to damp air for some time, loses this property and converts starch into lactic acid, (Boutron and Fremy.*)

Mitscherlich† thinks that fermentation is produced by cryptogamic vegetation, and putrefaction by infusoria. During winter, he has observed in putrefying substances, even when immediately surrounded by a warm atmosphere, the presence of infusoria only. These consist of globules ranged beside each other, sometimes to the number of twenty ; their diameter is about ·00 m. m. The other infusoria occasionally found, he considers as accidental. A certain amount of oxygen is necessary for the development of these vibriones. They are widely diffused throughout the intestinal canal, as well as in the buccal cavity and the stomach, and are contained in vast numbers in the matters collecting in the cavities and between the teeth, but he has never known them to be found in the blood, milk, urine, bile, or other fluids of that nature. When sugar is added to a liquid containing these vibriones, they multiply rapidly, a vegetation being formed at the same time ; but if the sugar is in very large proportion, it stops their growth, and fermentation sets in. Dr. Allen Thompson‡ states that "the embryos or earlier forms of various parasites and the ova of others, have been found in considerable numbers in the circulatory blood of various animals." The reader is referred to Robin's "Vegetal Parasite," for further description of vegetable growths in the human body. Blondeau considers all fermentations are preceded by the development of vegetable germs of various species, and which increase or remain inactive, according to the nature of the medium in which they are found, and whether it is favorable to their growth or otherwise. Each of these vegetations is capable of determining a peculiar fermentation ; they have nearly all the same composition, consisting of carbon, hydrogen, oxygen and nitrogen, nearly in the proportions in which they exist in albumen. The carbon is derived from the decomposition of carbonic acid, the hydrogen and oxygen from water, and the nitrogen from ammonia. These substances are furnished by the azotized and non-azotized mate-

* Sur la fermentation lactique, Journ. de Pharm. t. 27, 1841.

† Journal de Pharmacie t. 4, 3e. s 1843.

‡ Todd's Cyclopedia Anat. and Physiology, Article Ovum.

rials present in the liquids, and which consequently suffer decomposition. The acids formed in fermentation are frequently higher oxides (i. e. contain more oxygen) than the neutral substances (starch, ete.,) the growth of the cryptogamic vegetation being always at the expense of the carbonic acid and the ammonia. The plant appropriates the carbon and hydrogen and rejects the oxygen, which unites with the hydrogen of the ammonia or oxi izes one or more of the neutral bodies present, forming an acid or is evolved in the gaseous form, which frequently is the case in the lactic and butyric fermentations ; when mannite is for ed, the evolution of hydrogen ceases. When the plant has perfected its condition in the globule state, it rises to the surface in order to complete its existence and reach its full fructification. The liquid now becomes clear, and is covered with a white or colored dust, formed by the accumulation of the sporules of the cryptogamia. The lactic fermentation is caused by the growth of the Penicillium glaucum ; the acetic, by the Mycoderma vini, or the Torula aceti. The latter are described as ovoid globules, resembling the Torula cerivisiæ, or yeast plant, but distinguished therefrom by forming voluminous membranes, consisting of these globules united together ; the T. cerivisiæ never uniting to form membranes.

The membrane that covers putrid urine is principally composed of the Penicillium glaucum ; its development being in the ratio of the conversion of urea into carbonate of ammonia. When the mould on the surface of putrid urine is placed in contact with fresh urine, it causes it to become cloudy at once, and entirely converts the urea into carbonate of ammonia within 24 hours, instead of requiring eight days, as under other circumstances (Blondeau.)

Liebig* states that when fresh urine is enclosed in a clean vessel with air, oxygen is absorbed and a certain quantity of urea decomposed; with the disappearance of the oxygen the decomposition ceases, to be renewed by the access of fresh air, and so on, until the urea is finally all converted into carbonate of ammonia, a small quantity of acetic acid being formed at the same time. The greyish white deposit found in putrid urine produces the rapid spoiling of fresh urine, even without access of air,

*Liebig's Handwörterbuch.

(probably only to a limited extent.) Gmelin* observes that when urine is enclosed in a glass tube and heated to 212 F. and allowed to cool, the conversion of urea into carbonate of ammonia takes place as in open air, but without any appearance of fermentation. Prussic acid also decomposes under the same circumstance, as it does in air. It is most probable that these changes are induced in the urine, by the small amount of oxygen retained by it, notwithstanding the temperature to which it is exposed.

Another class of decompositions commonly placed in the list of fermentations is produced by a very limited number of organic substances; these substances are diastase and synaptase or emulsin. These ferments, if they are entitled to that name, possess a chemical composition and structure similar to, if not identical with gluten. There is indeed reason to believe that they are but gluten in a peculiar state of hydration. This can only be a conjecture, since there is no means of obtaining either gluten or these agents in a state of such absolute purity as to yield dependable results upon analysis. (Fownes.†) Diastase is contained in malted barley and other grains, being formed from gluten in the act of germination, and is the cause of the property possessed by malted grains of converting starch into grape sugar; the same effect can be produced by other means; thus starch mixed with water to a paste and excluded from the air is converted in a few weeks into grape sugar, but when in contact with gluten at 140° F. in eight hours; when the gluten exists in the form of diastase, in one hour.‡ The same effect is produced by boiling starch made into a thin paste with water, to which about 5 per cent. of sulphuric acid has been added. Lignin or woody fibre is also convertible into sugar by a long continuance of the same process. Starch paste, when long boiled, becomes thin and sweetish; this is caused by a small quantity of gluten being retained by the starch in the process of manufacture.§ A fresh aqueous extract of malted grain converts starch rapidly into sugar; when left exposed to the air for some time, it becomes cloudy,

*Gmelin's Handbuch.

† Journal of Pharmacy, 1843.

‡ Gmelin's Handbuch.

§ Liebig's Handwörterbuch.

a flocky deposit forms in it, and if grape sugar is now added to it, the alcoholic fermentation sets in briskly. If left still longer exposed, it becomes strongly acid and has a putrid odor; it now rapidly converts sugar into lactic acid. Bitter almonds contain a peculiar crystallizable principle called Amygdalin; when this is mixed with the albumen or gluten of the almond, in presence of water, the amygdalin is decomposed into hydrocyanic acid, oil of bitter almonds (hyduret of benzoyle) and grape sugar. This albumen or gluten differs somewhat in its properties from the forms met with elsewhere; the name synaptase or emulsin has been applied to it. The gluten of other grains, diastase, etc. produces the same effect in a lesser degree. Emulsin also effects the decomposition of salicin into saleginin and sugar, and the conversion of the salts of myronic acid (contained in black mustard seeds) into the volatile oil of mustard, to which its pungent properties are due. In all of these decompositions one or more atoms of water, enter into union with the products of decomposition; its presence, therefore, is essential.

Exposure to a temperature of 212° F., the presence of alcohol, ether, and many chemical reagents entirely destroy the properties of diastase and emulsin.

Of all the various phenomena presented by putrefaction and fermentation, none have obtained the tithe of the share of attention that has been paid to the progress of the alcoholic fermentation. This process, so important from its general application in domestic life, as well as in the manufacture of various commercial products, excited the interest of investigators at an early date. In 1680, Leuwenhock discovered the globular character of yeast, but did not attribute vital properties to it. Desmaziere, in 1826, furnished a more accurate account of this substance, but erred in describing it as infusorial, under the name of the mycoderma cerivisiæ, Desmaz. The credit of finally establishing the true nature and properties of the yeast plant is due to Cagnard Latour, who, a few years later proved that the conversion of sugar into alcohol and carbonic acid was caused by the presence and growth of the Torula cerivisiæ, a cryptogamie plant, existing only in the form of globules.

The nature of yeast is thus described by Blondeau.* There

* Journal of Pharmacie, t. 12, 3e. s. 1847.

are two species of germs present in yeast, those of the Torula cerivisiæ and those of the Penicillium glaucum. The germs of the first (the true yeast plant) multiply with great rapidity, but never form stems or deviate from the globular condition. The P. glaucum also multiply at first in globules, but they soon extend themselves, unite and form an arborescent vegetation. The germs of the P. glaucum are much smaller (1-400th m. m.) than those of the T. cerivisiæ (1-100th m. m.) so that they can readily be separated by filtration, the P. glaucum passing through the filter while the larger globules of the Torula remain behind. The liquid that has passed *through* the filter, when placed in contact with sugar and water, develops the ramifications of the Penicillium glaucum, and the lactic acid fermentation commences. The globules remaining *upon* the filter when also placed in water and sugar, immediately produce the alcoholic fermentation. These remarks agree with the observations of Andral and Gavarret,* who found that "when yeast is mixed in a deep glass vessel with water, and allowed to remain at rest for some time, it separates into two portions, one of which rises to the top, whilst the other sinks to the bottom. That at the top, consists of the P. glaucum, and when placed in sugar and water induces the lactic fermentation. The powder collected at the bottom, is the yeast plant, and when placed in sugar and water determines the alcoholic fermentation at once." Yeast, as commonly obtained from the brewers, is a viscid, frothy, pasty mass, of a whitish yellow color, a sour smell like spoilt beer, and a bitter taste. When separated from the excess of fluid by pressure or drying, it becomes hard and brittle, still preserving its globular construction when viewed under the microscope, and if not exposed during the process to too high a heat, will still excite the alcoholic fermentation and is in fact an article of commerce particularly in Germany, under the name of "pressed yeast." The property of exciting fermentation, possessed either by fresh or by pressed yeast, is easily destroyed in many ways. A temperature approaching the boiling point, all powerful chemical agents, washing with alcohol, ether, and even (if long continued) by pure water itself, completely check its action. When yeast is rubbed upon a slab with a muller until the globules can no longer be

* Annales de Chim. et de Physique, 1843.

seen, it loses its power of inducing the alcoholic fermentation, (Dr. Ludersdorf*), but according to Dr. Schmidt, of Dorpat,† will still cause the lactic fermentation in a suitable liquid. The bags containing pressed yeast, require much care in handling them; if they receive blows or are permitted to fall from a height, the part injured becomes soft and sticky, changes color, and loses its power of producing fermentation, and in a short time becomes putrid.‡ An extreme degree of cold does not appear to injure the properties of yeast. Cagnard Latour§ states that it still retains its fermentative power, after having been subjected to a temperature of —78° F. produced by admixture with solid carbonic acid. When yeast is triturated with sugar it does not dissolve as erroneously stated by Dobereiner,‖ but loses its opaque whiteness, and forms a yellowish semi-transparent liquid, in which the globules can still be readily detected by the microscope; they are, however, much smaller, and are incapable of inducing the alcoholic fermentation. The sugar is at the same time converted into uncrystallizable sugar. Yeast that has been treated with alcohol or ether, still preserves its appearance, but will not excite fermentation, the portions remaining undissolved consisting of the external cortical part of the globule, the skeleton, as it were, of the part. The living globule consists of a non-azotized insoluble sheath, possessing the chemical composition of cellulose, ($C_{24}$ $H_{21}$ $O_{21}$) and resisting the action of most solvents and chemical agents, and of an internal azotized substance, very similar in composition to protein. (Mulder.¶) According to the results obtained by Mitscherlich,** however, it differs essentially from the protein bodies in not containing any sulphur, the ashes consisting almost entirely of the phosphates of potassa, lime and magnesia. Schlosberger†† maintains, on the other hand, that sulphur *is* contained in small amount. The experiments of Mitscherlich appear worthy of the most dependence, having been apparently conducted with great care. In most of the

* Chemical Gazette, 1846.

† Liebeg's Handwörterbuch.

‡ Pharmaceutical Journal, 1849.

§ Annal. de chim. et de Physique, t. 68, 1838.

‖ Journal de Pharmacie, t. 1.

¶ Chem. Gazette, 1845, and Löwig's Organic Chemistry, Amer. edition.

** Chemical Gazette, 1846.

†† Pharmaceutical Journal, 1846.

examinations of yeast, it is probable that the mixture of the Torula cerivisæ and of the Penicillium glaucum (the common yeast of the brewers) has been used, yielding, of course, inacurate results. Liebig observes that, when yeast is washed on a filter for a long time with cold distilled water, deprived of air, and always covered with a layer of water, a residue is finally obtained no longer capable of effecting a solution of sugar. The washing water, however, acquires this property, but soon loses it when exposed to the air. Probably the lactic fermentation is induced by the presence, in solution, of part of the protein contents of the globules. The alcoholic fermentation will not take place under these conditions. When yeast that has been boiled in water (to destroy its vitality) is exposed to the air in an open vessel, it acquires in 12 days a dull yellow color, and a sharp disagreeable odor, still retaining an acid reaction. The globules retain their usual appearance under the microscope, excepting that their surface becomes less uniform, somewhat resembling shagreen. Within a month and a half, the mass becomes greenish-brown, and has a strong odor, like Gruyere cheese, with a slight acid reaction. Even after five months' exposure to the air, it does not dry, but forms a brown earthy paste, and is covered with mould, has a putrid odor and alkaline reaction. When mixed with water, it appears under the microscope as being composed of an infinite number of small irregular points or black grains, mixed with very pale globules, still resembling those of yeast."* The mould here spoken of is formed by the well developed stems of the Penicillium glaucum, which always forms on yeast when exposed for a length of time to the air, as it does on most other nitrogenized bodies. The globules spoken of above are only the outer cellular envelop of the yeast globule, and are devoid of all its characteristic properties. The course of the fermentation and growth of the yeast plant, and consequent duration of the fermentation, depend very much upon the nature of the liquid in which it is placed. When yeast is mixed with a solution of sugar containing nitrogenous substances, (the protein bodies, etc.,) as for instance wort, or infusion of the malted grain, the fermentation soon sets in, proceeds vigorously, and is soon completed. The yeast is found to have increased eight times

* T. A. Quevenne. Jour. de Pharm., t. 24. 1838.

over the quantity originally added; and this is the case, no matter how frequently repeated. But when, instead of a mixed solution of nitrogenized matter and sugar, a pure solution of sugar is taken, and mixed with two or three per cent. of yeast, the march of the phenomena is widely different. The action is vigorous at first, but soon slackens, and long before the sugar is exhausted, ceases entirely. If the weight of the yeast is accurately determined by drying a portion and weighing it, it will be found that during the brisk action the yeast increases in weight from 10 to 20 per cent., but decreases again as the fermentation proceeds, until at its close the weight is rather less than at the commencement of the experiment. (Quevenne.*)

During the progress of the alcoholic fermentation, the usual products are carbonic acid and alcohol, in the proportions of 44.8 and 47.2, but under certain circumstances other compounds appear, the formation of alcohol decreasing in a corresponding ratio; thus M. Filloy† remarks that "occasionally in the fermentation of molasses from the beet root sugar manufacture, when the molasses, either alkaline or acid, is mixed with five or six times its weight of water, and yeast added, the fermentation proceeds for a time, when nitrous acid suddenly appears, and the fermentation immediately ceases; this can be prevented by the addition to the molasses, diluted with twice its bulk of water, of 3 or 4 per cent. of sulphuric acid, and boiling it for a few minutes. Neither nitrous nor nitric acids exist in the syrups previously to fermentation, the nitrous acid being formed during the decomposition." This is corroborated by an observation made to the author by the manager of one of the largest distilleries of rum, from molasses, in New York, that frequently a gas is evolved from the fermenting vats that violently attacks the eyes, so that it is almost impossible to remain in the vicinity of the fermenting liquid. This, doubtless, is nitrous acid, and could probably be prevented by the same treatment with sulphuric acid. Yeast boiled in water and filtered, causes the viscous fermentation in a solution of sugar; a substance much resembling gum being produced with the evolution of two volumes of hydrogen with

* Jour. de Pharm., t. 28. 1841.

† Sur la production du Gas Nitreuse pendant la fermentation. Jour. de Pharm., t. 12, 1826.

one volume of carbonic acid. The same effect is produced by water in which gluten has been boiled. (Defosses.*)

When an infusion of nutgall is added to a solution of sugar in alcoholic fermentation, it at first retards the action, but soon resumes its activity. When the fermentation is ended, the tannic acid is found to be converted into gallic acid. (Lacroque.†) Brandecke states that the alcoholic fermentation can be induced in a solution of grape sugar containing tartrate of ammonia, by the addition of the most discordant substances, such as clean straw, pure charcoal, asbestos, flowers of sulphur, etc. Dopping and Struve, and Trautscholk† repeated the experiments, but found no alcohol was formed, though gas was evolved. Bone-black digested with the solution and then removed by filtration, deprived the liquor of its fermenting property, itself acquiring it when retaining a little sugar. Trautscholk considers, therefore, that the effects produced were due to nitrogenous impurity in the sugar, removed by the bone-black. When yeast is successively placed in contact with three separate portions of sugar and water, it produces complete fermentation and removal of the sugar in the first liquid, but partial fermentation in the second, and scarcely any action whatever in the third. (Quevenne.) Liebig remarks† that, "according to the experiments of Thenard, 20 parts of yeast, after being completely fermented, left 13.7 parts of insoluble residue. This being again placed in contact with a fresh portion of sugar, was reduced to 10 parts. This latter residue was white, and presented all the properties of lignin. It exerted no further action on sugar.

Besides the admixture of the germs of the Penicillium glaucum in common yeast, Mitscherlich‡ thinks two species of yeast can be clearly distinguished; "these are the so-called "*Upper and Lower*" yeast of the (German) brewer's. The former increases at a temperature between 32° and 45° F.; this is the yeast of the Bavarian or Lager beer. The upper yeast grows at a temperature of 77° F. and is the best developed. The lower yeast consists of globules of different sizes, always separate; a small globule has never been observed attached to a

*Jour. de Pharm., t. 15. 1829.

†Liebig's Handwörterbuch.

‡Journal de Pharmacie, 1843.

larger one ; the little ones are always isolated. In the upper yeast, the small globules are seldom separate, being attached to the large ones, forming chaplets, etc., these globules augment by forming buds, (growth by gemmation.) The lower yeast, on the contrary, increases by little globules isolated in the liquid. In the old yeast, an envelop and granular contents can be readily perceived in the globules, and by compression these can be made to burst and discharge their contents." He thinks that in the lower yeast, the globules burst and each granule gives rise to a globule, being thus reproduced by spores.

The temperature most favorable to the alcoholic fermentation is from 68° to 73° F., the production of alcohol being then the greatest ; the action being very slow and irregular at lower temperatures. If, on the other hand, the temperature becomes too high, very little alcohol is formed, although the evolution of carbonic acid continues. Quevenne* states that "when fermentation is induced in a solution by yeast, and the temperature very gradually raised, the evolution of gas becomes more rapid as the heat increases, until at 122°F. no alcohol whatever is formed, the amount produced decreasing in proportion as the heat approaches that point ; the formation of gas does not then cease, however, but continues to be rapidly formed, even when the liquid reaches the boiling point, and so continues for three quarters of an hour or more, when it ceases forever. The product of this fermentation carried on at 212° F. is a substance soluble in water, (from which phosphate of lime separates,) insoluble in alcohol and ether, reddens litmus faintly, gives precipitates with perchloride of iron, acetates of lead and copper, proto-chloride of tin, nitrate of silver and tannin. Oxalate of ammonia scarcely troubles it ; when heated, it burns without flame and leaves a hard voluminous charcoal, it contains nitrogen and the phosphates of lime, magnesia and of the alkalies. The substance resembles, but is not identical with humus, the residue formed by the decomposition of wood, etc., in the earth." The author has been informed by a gentleman possessing great experience in the processes of fermentation, that in the fermentation of molasses, if the temperature exceeds 90° F., the yield is so much decreased that the process becomes

*Journal de Pharmacie, t. 28, 1841.

unprofitable. It is probably owing to this cause that so much uncertainty exists with regard to the advantageous management of the fermentation of grain, etc., some distillers being able to obtain a far greater product than others.

Alcoholic fermentation in common with all the varieties of decomposition and putrefaction, is retarded and even entirely prevented by many causes. Gay Lussac first directed attention to the fact, that when sound ripe grapes are passed up into a tube closed at top and filled with mercury, (the air having been perfectly removed by carbonic acid,) and then crushed without access of air, the juice (or must) thus obtained, remains unchanged and without any appearance of fermentation; but if a single bubble of oxygen gas is permitted to enter, the juice quickly undergoes the alcoholic fermentation.*

The presence of atmospheric air or oxygen appears essential to the first development, if not to the continuance of nearly all forms of decomposition. Thus milk placed in a close vessel which it entirely fills, and boiled therein to expel all remaining air, and then hermetically sealed, may be preserved sweet for an indefinite length of time. Meat, vegetables, and indeed, most organic substances can be kept in the same manner for years. Eggs lose their property of absorbing oxygen by immersion in milk of lime, the small amount of carbonic acid contained within the shell uniting with the solution of lime that penetrates into the pores of the shell and forming an insoluble carbonate, choking up all the apertures by which air can enter. Eggs have been found sweet after being kept in this manner over 300 years.† Wood sunk several feet beneath the surface of a peat bog is preserved from decay, the oxygen absorbed by the organic matter above it, not being able to reach it. The same cause explains the preservation of human bodies occasionally found in these bogs. A temperature below 32° or above 212° F., prevents the commencement and interrupts the progress of nearly all fermentations and putrefactions. Thus meat or vegetables, while frozen, will keep for centuries, merely losing by evaporation the water they contain if exposed to the air, but when placed in a suitable temperature, decomposition commences as usual. If milk, meat,

* E. Julia Fontenelle, Jour. de Pharm., 1823.

† Gmelin's Handbuch.

grape juice, etc., are daily heated to 212° F. for a short time, fermentation will be indefinitely postponed.* M. Payen,† who was appointed by the French Government to examine into the cause of the sickness produced in the army by mouldy bread, found that the poisonous properties were caused by the presence of several forms of cryptogamia, principally the Oidium aurantiacum that attacked the bread, and destroyed the starch and other constituents of the flour in obtaining the elements for its nourishment; he found that the germs would again vegetate after being exposed to a temperature of 248° F., but were destroyed by a heat of 284° F. This is a remarkable instance of the tenacity of vegetable life.

The removal of water, either by desiccation or union with some substance that retains it with great force, (as sugar, salt, etc.) also prevents all change, as instanced in the preserving of fruits, salting of meat, drying of plants, fruits, meats and many other examples in domestic life. The action of some chemical agents belongs to the same class, but more generally their efficacy is due, either to their entering into combination with the organic substance, as in the coagulating of albumen by creasote, and probably in the union of the former with corrosive sublimate and other substances of that nature, or by the chemical used, acting as a poison upon the cryptogamic and infusorial organisms that are generally associated with putrefaction and fermentation.

Dr. John H. Brinton states in the Medical Examiner, July, 1854, that he succeeded in preserving, for over 60 days, fresh meat and anatomical specimens, such as muscular and nervous tissue, from all change excepting a slight bleaching of the muscles, simply by covering the specimen, by means of a brush, with a solution of gutta percha in benzole. The anatomical preparations were first injected with solution of chloride of zinc, or with arsenic. The meat received, however, no previous treatment. This process acts, in all probability, not simply by the exclusion of oxygen, but also by the impregnation of the tissue and consequent destruction of existing germs of infusoria, etc., by the vapor of benzole.

Mitscherlich‡ observes that those chemicals that destroy the life

*Gmelin's Handbuch.

†Annales de Chim. et de Physique, 1848.

‡Journal de Pharmacie, 1843.

of the fungi, stop fermentation, while those that only destroy animal life are without action. The volatile oil of mustard completely prevents the fermentation of grape juice. The volatile oils of peppermint, aniseed, turpentine and many or all others are without effect.* The spoiling of wine, (la poulle or la graisse,) which is caused by or accompanied with the production of the Penicillium glaucum, is prevented by the addition of sulphurous acid; this is due, in Defosse's† opinion, simply to its presence as an inorganic acid, and not to its affinity for oxygen. If sulphuric acid is added instead, it decomposes the tartrate of potash present, setting tartaric acid free, being itself removed from the solution; this does not take place with sulphurous acid. Quevenne states that creasote, oil of turpentine, the mineral acids, oxalic and prussic acids, when added in the proportion of 6 *grs.* (?) to 20 grammes of sugar, all prevent fermentation. Arsenious acid, tannin, morphine and strychnine in the same proportions, exert no action. The alkalies rather retard it. Schwann‡ remarks that arsenious acid and corrosive sublimate stop putrefaction; extract of nux vomica stops the production of sulphuretted hydrogen and of infusoria, but does not prevent the occurrence of mould. Gilgenkrantz§ has observed a vegetation of the genus Leptomitus or Hygrocrosis growing in a solution of arsenious acid, and another species in solution of corrosive sublimate. These poisons consequently cannot prove injurious to the lower forms of vegetable life.

Helmholtz|| instituted a number of experiments upon the transference of the fermentative influence through membranes, etc. When fresh grape juice is placed in a test tube, the mouth tied over with bladder, and immersed with the bladder end downwards in grape juice in fermentation, the fluid in the tube remained unaltered, except acquiring by endosmose a slight odor and taste of the external liquid. Meat and water, similarly treated and immersed in a putrefying solution, acquired a putrid odor, with development of $CO_2$ and SH.; but instead of dis-

*E. Julia Fontenelle, Jour. de Pharm., 1823.

†Jour. de Pharm., 1829.

‡Poggendorf's Ann. 41, and Gmelin's Handbuch.

§Jour. de Pharm., 1837.

||Gmelin's Handbuch.

solving to a paste, as meat does under usual conditions, it preserved its structure, and became firmer than boiled albumen. Gelatine likewise appeared putrid, but without becoming cloudy. In none of these experiments was there any formation of mould or of infusoria. Löwig very properly remarks upon these investigations, that the apparent decomposition was not real, since there was no structural change, and that the smell and the presence of gases was entirely due to the endosmose through the membrane, no putrid change or fermentation having really taken place. Schwann* found that, when meat and water are boiled in a flask for some time and only air permitted to enter that has been passed through a red hot glass tube, neither infusoria nor mould occurred, and on opening the flask its contents were unchanged by putrefaction or fermentation; the same result was obtained with solution of gelatine and with grape juice, etc. On subsequent exposure to the air, infusoria soon appeared and fermentation commenced. Schultze† obtained precisely similar results by passing air through a solution of caustic potassa and through concentrated sulphuric acid, previously to its admittance into the flask, containing the boiled meat and water. His experiments were directed more particularly to the development of infusoria.

Schröder and Von Dusch‡ have lately given the details of experiments tried by them upon the effects produced by filtered air, upon fermentation, etc. They have established the fact, "that when air is passed through a tube filled with raw cotton, moderately compressed, it becomes incapable of inducing fermentation or putrefaction in substances that would rapidly undergo these changes if common air was substituted. Thus, meat, broth, wort, etc., were preserved for weeks in flasks, in which they were boiled, a constant current of filtered air being drawn through the flasks. No change of any kind was perceptible, even in summer weather. When milk was tried in the same manner, however, it became sour nearly as soon as in the open air, thus indicating an essential difference in the principles involved in the respective decompositions." The author

* Poggendorf's Ann. 41, and Gmelin's Handbuch.

† Edinburgh Philosophical Journal, 1837.

‡ Liebig's Annalen, 1854, and Medical Examiner, June, 1854.

has himself repeated the experiment of preserving boiled meat and water in a flask, having an aperture of at least one inch diameter, closed merely with a plug of raw cotton, part of the cotton being formed into a ball, surrounding the neck of the flask and confined with a thread, to prevent the passage of air between the sides of the aperture and the plug of cotton. Meat broth, thus prepared, was found to be perfectly sweet and unchanged in every respect, after the lapse of six weeks in the months of June and July; a portion of the same broth placed in a bottle with a glass stopper, became so offensive on the third day as to require its removal.

These results, above mentioned, appearing to establish the theory, that all fermentations, etc., are induced by the presence in the air of the germs of organic life, led the author to make the following experiments, with the two-fold purpose; 1st, of deciding whether this property possessed by cotton was peculiar to it alone and due to its structural arrangement, or whether it was common to it and to all other finely divided substances; and 2d, the hope of detecting in the air these invisible germs, or at least of obtaining satisfactory proof of their existence. The apparatus made use of was essentially that of Schröder and Von Dusch, with merely such alterations as the purposes in view required. It consisted of the flask *a*, of about one quart capacity, in which was placed the liquid experimented on, closed tightly with a cork, through which passed two glass tubes, one connecting with the five gallon tin cannister *b*, the communication with which could be intercepted at pleasure by a stop-cock, the other leading to the lower end of the filter tube *c*, a glass tube, 1¼ inches in diameter and 18 inches long, closed at both ends by corks; a diaphragm of fine copper wire gauze was placed a little above the lower cork; through the upper cork was inserted the bent limb of the drying tube *d*, containing fragments of dried chloride of calcium, the other end of the drying tube, connected with the nitrogen bulb or washing apparatus *e*, containing about ¼ ounce of water; from this a tube passed to the bell glass *f*, through the cork at top, through which also passed another straight tube *k*, reaching to the centre of the bell glass, for the purpose of admitting air. The bell glass was closed at the bottom by a plate of ground glass *g*, supported upon a sliding support *h*, kept at any desired elevation by a set screw; within the bell glass was placed a beaker glass *i*, resting upon the glass plate *g*, so that it could be removed at pleasure. Into this beaker glass was poured an infusion of malt, (wort,) similar to that in the flask *a*. The tin cannister *b*, was provided with an opening through which it could be filled and capable of being closed air tight; at the bottom was a discharge cock *l*, leading into a suitable receiver. It is evident that, if the joints of the apparatus are all closed air tight, (the cannister being filled with water,) and the cock *l* opened to permit the escape of the water, a current of air must enter through the tube *k*, into the bell glass *f*, and from thence pass through the washing tube *e*, the drying tube *d*, the filter tube *c*, and the flask *a*, containing the experimental liquid, and finally into the cannister *b*, supplying the void created by the escape of the water. The substance selected for the filtering medium was pure white sugar, in grains of the size of fine sand, all coarser and finer particles being removed by sieves of different sizes. It was chosen as being better adapted to this purpose than nearly any other substance, being readily obtained pure and clean, possessing an uniform composition, ready solubility and absence of color; it could also be heated to 212° F. without injury to its physical properties. The washing tube and water were used to prevent any organic matter, dust, etc., being mixed with the sugar in the filter tube, the presence of which might lead to errors in the subsequent examinations. The air was dried after leaving the water by the chloride of calcium, in order that no possibility might exist of the germination of the sporules favored by the moisture, that would otherwise be carried into the sugar, and which, if it had taken place, might cause them to vegetate, and thus transmit the germs from particle to particle, until finally carried over by the air into the flask *a*.

On the 30th Nov., 1854, ¾ pint of warm ale wort, fresh from the brewery, was put into the experimental flask *a*, and about six ounces of the same into the beaker glass *i*, about the same quantity also poured into a bottle and left exposed to the air. The filter tube was now filled with the sugar, previously heated in an air bath to 212° for 30 minutes. The joints being now made air tight, the contents of the flask *a* were brought into ebullition, which was continued until the tubes leading to the filter and to the cannister were heated throughout. The stop-cock *l* was now opened, and the water permitted to escape drop by drop, the air entering through the tube *k*, and passing through the whole apparatus to replace it. The water was allowed to run out at the rate of four gallons in 24 hours, being renewed once a day; of course the same quantity of air would pass through the flask *a* within that time; the temperature of the room 65° F. On the 4th of December the liquid in the open bottle was covered with bubbles, and a very thin pellicle formed on the surface. The contents of the flask *a*, and of the beaker glass *i*, remained unaltered. Dec. 5th. The liquid in the beaker glass showed a little mould on the surface. Dec. 6th. The mould was very perceptible in the beaker glass; a few globules were visible in the liquid when under the microscope. Dec. 9th. The liquid in the beaker glass had apparently passed through the alcoholic fermentation and become very acid, smelling strongly of vinegar; it was full of vibriones and covered with a thick mould. The experiment flask *a* remained perfectly clear and with no appearance of mould or fermentation. The air was drawn constantly through the apparatus, in the manner described, from the 30th of November until the 23d of December. It was then allowed to remain at rest until the 25th of January, 1855, no appearance of decomposition having occurred in the flask *a*; the washing and drying tubes *e* and *d* were disconnected, so that the air entered from the atmosphere immediately into the filter tube; this was done to make certain that the effects produced were not caused by the water or by the chloride of calcium. The air was now again drawn through as before, on alternate days, until February 26th, when the operation was concluded. On examining the water in the washing vessel *e*, it was found to be reduced by evaporation to about one drachm; it was perfectly colorless and transparent; suspended in it, however, there was a flocculent mass of the size of a pea, colorless and resembling very fine raw cotton; it was possessed of great tenacity, although of the most delicate structure. Under the microscope, the liquid was found to be filled with extremely minute circular globules, requiring great attention to distinguish them, resembling the globules of the Penicillium glaucum, although much smaller. The flocculent mass above mentioned was resolved into a vegetation of the utmost beauty and regularity, consisting of extremely delicate fibres interlacing with each other and covered in parts with sporules and globules. The plant resembled somewhat the Penicillium glaucum, but was far more delicate in its structure, and did not appear composed of globules extended longitudinally, forming cells, as is the case in the latter.

When a portion of the clear washing liquid was placed in a bottle with 10 per cent. of fresh sugar, a new flocculent deposit formed in a few days, possessing the general characteristics of that above described. A portion of the sugar from the top of the filter was next examined, but the most rigid scrutiny failed to detect any organic structure, either in the sugar in grains or in the solution obtained by dissolving it in 10 times its weight of distilled water. Another portion was then dissolved in the same amount of water, and placed aside for several weeks, but no trace of globules or other organisms could be found. The examinations and drawing were all made with a Powel & Leland's microscope, $\frac{1}{8}$ in. objective, and highest power of eye piece, giving a magnifying power of about 800 diameters.

When the flask *a* was opened, on the 26th of February, it presented all the properties of the original wort, being perfectly sweet, having a very slight acid reaction, and the odor and taste of fresh wort. On being subjected to distillation, the distillate obtained was neutral, possessed the character and smell of fresh wort, and, by treatment with bichromate of potash and sulphuric acid, proved the entire absence of alcohol. During the whole time this experiment was proceeding, the temperature was never below 65°, sometimes 75°, averaging over 70°.

At the completion of this experiment, a small clean bottle was filled with the still sweet wort from the flash *a*, and tightly corked. The next day it was found covered with froth and in brisk fermentation. The remaining contents of the flask were left therein, and closed with the cork through which the two glass tubes passed, thus affording an uninterrupted communication with the atmosphere through the tubes. At the end of the week the liquid was but little changed, having merely a musty smell; no mould was perceptible. The flask was now agitated, to expel the air contained therein and replace it with fresh air; the next day it was found covered with a thick growth of Penicillium glaucum, was strongly acid, had a putrid odor, and underwent rapid putrefaction.

On the 23d of November, one week previous to the above mentioned experiments, a portion of wort was placed in the apparatus, arranged as just described, the conditions being exactly the same as in the last experiment, excepting that the sugar was not heated previously to being placed in the filter tube. On the fourth day of the operation fermentation commenced, alike in the beaker glass and in the experimental flask *a*; a thick formation of mould covered the liquid; the process was then interrupted and commenced anew as previously described.

This result clearly indicates that there is contained in the sugar, as met with in commerce, a substance capable of being taken up by a current of air passing over or through it, and possessing the property, while thus suspended or dissolved in the air, of producing fermentation and the growth of mould in fresh wort; this property, however, being destroyed by a temperature of 212° F. The author believes that sugar possesses this property in common with all matter, organic and inorganic, that is not destructive to vitality. The action of cotton is due, therefore, simply to its finely divided condition, and not to any peculiarity in form or composition. The germs floating in the air being deposited or taken up again, precisely as finely-divided dust would be under the same conditions.

It is not probable that the globules found in the washing water are the germs of the plants causing fermentation, etc. It is more likely that they are the partially-developed globules, having a magnitude many times greater than the actual germ that is suspended in the air and distributed through all nature. The cryptogamia found in their full development, and the innumerable globules present had doubtless been nourished and attained their present size by the volatile matters given off by the fermenting liquids in the beaker glass *i*, and a portion of which must have been absorbed in theirpassage through the water. The true original germs are without doubt contained in the sugar in the filter, but being absolutely without nitrogeneous materials for their growth, even when the sugar is in solution, they remain in their pristine state.

The facts and experiments related in the preceding pages constitute the substance of nearly all that is known concerning fermentation and putrefaction, the study of which could cast any light upon the principles involved in these decompositions. Many experiments and observations have of course been made, that are not alluded to here, but none that the author could meet with have been omitted, that, in his opinion, could contribute to the knowledge of the subject or to the support of any theory.

Various views have been held by distinguished writers, upon the causes inducing fermentation and putrefaction. The only theories that need occupy our attention, however, are:—

1. The doctrine of catalysis, promulgated by Berzelius.

2. The doctrine of atomic disturbance, promulgated by Liebig.

And 3d. The doctrine of the agency of vital organisms in the forms of infusoria and cryptogamia, proposed by Schwann.

To which may be added the influence of ozone, as suggested by several writers, but which may be dismissed with merely the remark that it can only produce effects greater in degree, but similar in kind to common oxygen, since the presence of oxidizable matter instantly causes its disappearance, and the oxidation of the substance submitted to its influence. It is, doubtless, an important agent in atmospherical purification, but scarcely so in the class of decompositions now under notice.

The doctrine of catalysis consists in the assertion, that certain chemical changes are induced in bodies by the mere presence of other substances, these latter substances either undergoing no change whatever, or suffering a decomposition similar in kind to that of the other body present; as, for instance, the decomposition of peroxide of hydrogen by oxide of silver, the silver being reduced to the metallic state, with the formation of water and evolution of oxygen, both from the oxide of silver and the peroxide of hydrogen. The same effect being produced by the addition of finely divided metallic substances and by many oxides which do not suffer decomposition like oxide of silver. No satisfactory explanation has as yet been given of the cause of these peculiar changes, but there can be but little doubt that they are owing to the formation and subsequent decomposition of compounds, whose elements are held together by affinities so nicely balanced that the slightest cause is sufficient to disturb them. At all events, the doctrine of catalysis does not in any way assist us, since it merely gives a name to the phenomena, and substitutes a *word* in place of an *explanation*, (Gmelin.) It is unnecessary therefore to dwell longer upon this point.

The theory advocated by Liebig is substantially, that a ferment, or substance in fermentation, has its atoms in a state of change or motion, and that when this ferment is placed in contact with a body incapable of itself of entering into decomposition, like sugar for instance, it absorbs oxygen and imparts its state of molecular change to the sugar; those elements of the ferment being in motion, transmit a mechanical impulse to those of the body in contact with it, resulting in the disruption of the original structure and the formation of new compounds, as alcohol and carbonic acid. This is evidently the catalytic doctrine with the addition of the theory of a mechanical impulse to explain its modus operandi. Gmelin very correctly observes, that it is a mere *assumption*, the fact of a mechanical impulse communicated from the atoms of ferment to those of the sugar, without any *demonstration* whatever, and that if the possibility of this transference of motion be granted, it should according to analogy displace the total atom of sugar and not merely a portion of its elements. He further remarks that if mechanical disturbance is the cause of these changes, they should be, also, produced by agitation with sand or other finely divided substances; still more so by the decomposition, by sulphuric acid and zinc, of water in which sugar is dissolved, and also of the carbonated alkalies by acids in the same solution, in none of which cases is there the slightest disturbance of the atomic structure of the sugar (Gmelin's Handbuch.) It may be added that the experiments of Schwann, Schultze, Helmholtz, and Schröder and Von Dusch, are totally irreconcilable with such a theory.

The assumed facts upon which the doctrine of catalysis depended for its illustrations, are one by one being slowly removed by the advancement of science, and it will doubtless shortly be placed by common consent in the same category with the theories of Phlogiston, of the transmutation of metals, and with various others that have uselessly occupied the minds of men in all ages. Thus, the action of finely divided platinum in causing the conversion of alcohol into acetic acid, the decomposition of alcohol by sulphuric acid, into ether and water, and the fact that an alloy of copper, nickel and zinc is entirely soluble in diluted sulphuric acid (Liebig*) were placed among the most prominent examples of this law. But it has now been known for some time that the action of platinum black is due to its property of condensing oxygen and other gases within its pores, thus increasing the affinities of oxygen for many other substances, a

* Annales de Chim. et de Physique, 1839.

process of which we do not know the rationale it is true, but the same may be said of nearly all chemical action. The conversion of alcohol into ether is at least as well explained by the formation and decomposition of sulphovinic acid, and certainly acquires no elucidation from the catalysis doctrine. It is within the last few weeks that Mohr's* paper upon the determination of copper has appeared, wherein he states that copper precipitated in the pulverulent form from an acid solution by iron, is soluble in a dilute solution of sulphuric acid, obtaining oxygen from the acid; its solubility being due to the state of its fine division, thus entirely doing away with the value of an experiment that was dwelt upon with peculiar emphasis by Liebig, as illustrating the action of this theory.

The views advocated by Schwann are much more in accordance with the results obtained of late by many investigators, and explain in the most satisfactory manner many points that are utterly at variance with the necessary deductions from the previous theories. In fact it may be considered as proved, *that the principal phenomena of putrefaction and fermentation are due to the presence in the air of the germs of infusoria and cryptogamia, which by their growth produce those changes with which we are familiar.* The objections made by Liebig to this theory are as follows:

1st. That the yeast of beer has not the composition of actual fungi, but of that of gluten. 2d. That it is not explained in what manner these microscopic beings produce these changes. 3d. If fermentation is produced by the growth of vegetation, it should not take place in a solution of pure sugar when yeast is added, since it contains no nitrogen or other elements essential to the plant. 4th. Fermentation is induced in solution of sugar by caseine or emulsion of almonds, without the production of vegetation. 5th. In a thousand places putrefaction takes place in cheese, blood, urine, etc. without the production of infusoria.†

To these questions the following answers may be given:

1st. That it is unimportant whether the composition of the yeast plant resembles that of fungi or not, since the question is not whether yeast is a fungus, but whether yeast is a plant.

*Liebig's Annalen, bd. 97, s. 392.

†Gmelin's Handbuch.

We *positively know* that the yeast globule *is* a plant, possessing a cellular structure, consisting of an external envelope resembling lignin, and of an azotized internal substance. 2d. That we are equally ignorant of the manner in which the inorganic constituents of the soil are converted into blooming flowers, yet, nevertheless, this conversion *does* take place. 3d. That fermentation only occurs in a solution of pure sugar when yeast *is* added, and then only in proportion to its amount. When the vitality of the yeast globule is exhausted, its remains serve as a soil or means of nourishment to a new crop of the plants, so far as they are capable of decomposing the structural arrangement of the dead globule, in the same manner that grass grows continually on the so called perpetual meadows, where the crop is never removed, but suffered to rot where it grew; but as the yeast globule is not entirely decomposed, the means of nourishment soon become exhausted and the growth ceases, and with it the fermentation ends. This view is supported by the fact, that yeast placed in a solution containing all the elements for its growth, as in worts for instance, increases eight fold, but when in pure sugar, weighs rather less at the end than at the commencement of the process. 4th. That caseine, etc. only induces fermentation when it has been placed in the air long enough to have the germs of these organisms deposited upon it, and even if exposed to the air, no change takes place if the air has previously been heated, passed through powerful chemical reagents, or filtered through cotton, sugar, etc. The souring of milk is an exception to this and requires a separate explanation. In every case excepting milk, globules of cryptogamia or infusoria *have* been found. 5th. Is sufficiently answered by the above remarks.

After a careful consideration of the various facts connected with this subject, the author is of opinion that these phenomena should be distributed into three classes; the action in each being distinct in its causes and manifestations. These are, 1st, eremacausis; restricting this term to those decompositions that are produced simply by oxidation without the presence of any substance, either organic or vital, other than those immediately concerned in the decomposition. The oxidation of oil, the rotting of wood under certain conditions, the formation of acetic acid from alcohol by means of platinum sponge, and many other instances, precisely similar in the principles involved to the rusting of a metal, or the slow oxidation of phosphorus, require no other explanation than is needed in all chemical processes.

2d. Under this head should be placed those changes that are induced in certain bodies by the presence of another substance, it determining the fixation of water and the formation of new compounds, as in the conversion of starch into grape sugar by diastase, the decomposition of amygdalin, etc. These may be looked upon simply as instances of double decomposition, in which the gluten or emulsin, parts with one or more elements of water to the body with which it is in contact, which it again assumes, when needed, from the water present in the solution; the gluten playing a part with the water similar to that performed by the oxide of nitrogen in the oil of vitriol chamber, or by acetic acid, in the old or Dutch method of manufacturing white lead. These decompositions should not be grouped with fermentation or putrefaction, but be considered, like eremacausis, as examples of mere chemical affinity.

3d. The last division of this classification contains all the processes of fermentation and putrefaction that are connected with the growth of cryptogamia or infusoria, and includes all those cases not embraced under the previous headings, in which the decomposition is produced by the presence of a fermenting or putrefying substance, by means of the germs of vegetation or animal life contained therein, or in the air, fluids, vessels, or other materials used. Those conditions, therefore, that destroy the vitality of the plant or animal, or remove it from the sphere of action, necessarily interrupt the decomposition by removing its cause, thus explaining the effects of heat, chemical agents, and the exclusion, heating and filtration of air. The immediate action exerted by the living germ in effecting decomposition is of course unknown to us; we can only state, that the affinity uniting the elements in combination is so slight, that many disturbing causes are sufficient to overturn the balance and resolve the compound, not into its elements, but into other groups having stability enough to remain permanent under such conditions. The plant needing part of the elements present in solution, perhaps the elements of water, withdraws them from the organic substance, in which they exist less firmly united than in water; the nascent combinations probably having the power to assimilate again the water from the medium present, either as water or its elements, thus furnishing the products of fermentation. This may be one reason why fermentation ceases with the absence of water.

Our knowledge of the subject is too insufficient to enable us, in all cases, to decide to which class certain decompositions belong, or whether, in some instances, all three of the above processes may not simultaneously exist. Thus, the souring of milk, when boiled and exposed to filtered air, may depend either upon the caseine existing in it, in a state similar to that of gluten in malted grain, only needing the absorption of oxygen to render it active, or from fresh milk containing germs, which like those of the Oidium aurantiacum, require a heat above the boiling point to destroy.

It is sometimes objected to this theory of vital action, that the existence of these germs in the air is neither proved nor probable, since it is unlikely that the air should contain such vast quantities as must be present if diffused in the manner indicated. To this it may be answered, that certain organic substances invariably are found covered with the same forms of mould and containing the same infusoria, when exposed for but a short time to the air; and since the doctrine of spontaneous generation has been proved, as Carpenter states, to be "without any claim whatever to be received as even a possible hypothesis," it must follow that all air holds in suspension the germs of these cryptogamia and infusoria, and if *this* is admitted the rest cannot be rejected. "John Marshall has detected the sporules of Uredo segetis near the apex of every grain, even in very fine samples of wheat, and which only wanted the influence of a cold and wet season, producing unhealthy action in the part, to develope themselves."* Carpenter further states, that "it is well remarked by Fries on this point, that the sporules are in such vast numbers, (in a single individual of Reticularia maxima he has estimated that 10 millions must be present,) are so subtle, (being invisible to the naked eye, except when collected in masses and appearing as a thin smoke, when diffused in vast multitudes through the air,)

* Carpenter's General Physiology.

are so light, (being raised perhaps by evaporation into the air, and are dispered in so many ways by winds, insects, elasticity, etc.,) that it is difficult to conceive a place from which they can be excluded."

In conclusion, the author earnestly calls the attention of *Physicians especially* to this subject, believing that it is only by the study of the phenomena of putrefaction, and by closely watching the varied appearances presented in the dissolution of organic remains, that the mystery and ignorance that now attend upon the causes of malaria, miasmatic and contagious influences, can ever be dissipated, and man placed in a position to grapple with what has ever been the scourge of his race.

*Philadelphia, April 16th,* 1855.

## Figures and Tables

**Figure f1:**
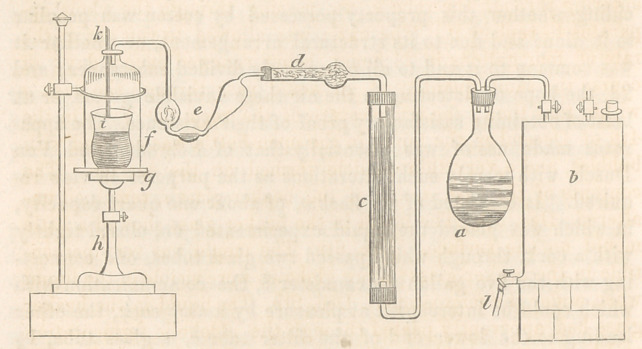


**Figure f2:**